# Activation of autophagy is involved in the protective effect of 17β‐oestradiol on endotoxaemia‐induced multiple organ dysfunction in ovariectomized rats

**DOI:** 10.1111/jcmm.13280

**Published:** 2017-07-17

**Authors:** Ming‐Tzeung Chung, Yen‐Mei Lee, Hsin‐Hsueh Shen, Pao‐Yun Cheng, Yu‐Chen Huang, Yu‐Ju Lin, Yu‐Yang Huang, Kwok‐Keung Lam

**Affiliations:** ^1^ Department of Obstetrics and Gynecology Tri‐Service General Hospital Songshan Branch National Defense Medical Center Taipei Taiwan, ROC; ^2^ Department of Gynecology and Obstetrics Taipei City Hospital Ren‐Ai Branch Taipei Taiwan, ROC; ^3^ Department of Pharmacology National Defense Medical Center Taipei Taiwan, ROC; ^4^ Department of Physiology and Biophysics National Defense Medical Center Taipei Taiwan, ROC; ^5^ Department of Pharmacology Taipei Medical University Taipei Taiwan, ROC; ^6^ Department of Anesthesiology Catholic Mercy Hospital Hsinchu Taiwan, ROC

**Keywords:** oestrogen, ovariectomy, endotoxaemia, septic shock, multiple organ dysfunction syndrome, oxidative stress, inflammation, autophagy, HO‐1, HSP70

## Abstract

Oestrogens have been reported to attenuate acute inflammation in sepsis. In this study, the effects of long‐term oestrogen replacement with 17β‐oestradiol (E_2_) on endotoxaemia‐induced circulatory dysfunction and multiple organ dysfunction syndrome were evaluated in ovariectomized (Ovx) rats. E_2_ (50 μg/kg, s.c., 3 times/week) was administered for 8 weeks, followed by the induction of endotoxaemia by intravenous infusion of lipopolysaccharides (LPS; 30 mg/kg/4 hrs). Oestrogen deficiency induced by ovariectomy for 9 weeks augmented the LPS‐induced damage, including endotoxic shock, myocardial contractile dysfunction, renal dysfunction and rhabdomyolysis. Cardiac levels of NF‐κB p65, iNOS and oxidized glutathione, free radical production in skeletal muscles, myoglobin deposition in renal tubules, and plasma levels of plasminogen activator inhibitor‐1, TNF‐α, and IL‐6 were more pronounced in the Ovx + LPS group than in the Sham + LPS group. Long‐term treatment of E_2_ prevented this amplified damage in Ovx rats. Six hours after LPS initiation, activation of the autophagic process, demonstrated by increases in Atg12 and LC3B‐II/LC3B‐I ratios, and induction of haem oxygenase (HO)‐1 and heat‐shock protein (HSP) 70 protein expression in myocardium were increased significantly in the Ovx + E_2_ + LPS group. These results suggest that activation of autophagy and induction of HO‐1 and HSP70 contribute to the protective effect of long‐term E_2_ replacement on multiple organ dysfunction syndrome in endotoxaemia.

## Introduction

Sepsis, which is the clinical syndrome of a systemic inflammatory response involving severe infection, is a main cause of morbidity and death in critically ill patients. A robust release of systemic cytokines, for example tumour necrosis factor (TNF), interleukin‐1 (IL‐1) and interleukin‐6 (IL‐6) is shown in the septic humans and in animal models 1, 2. Binding of toll‐like receptors by bacterial ligands, such as lipopolysaccharide (LPS), initiates the signalling pathway to activate NF‐κB, which triggers transcription of many important proinflammatory cytokines and chemokine genes 3. Substantial physiological changes may result in organ injury and multiple organ dysfunction syndrome (MODS). Several changes commonly seen in sepsis are tachycardia, hypotension, reduced cardiac ejection fractions, rhabdomyolysis, and acute kidney, lung and liver injuries 4, 5. Furthermore, severe sepsis is almost always associated with coagulation changes, frequently leading to disseminated intravascular coagulation (DIC) 6. Sepsis‐induced procoagulation is mainly due to the release of cytokines by endotoxins, which subsequently triggers the expression of tissue factors on monocytes and endothelial cells 7.

The inflammatory response in sepsis increases the activity of inducible nitric oxide synthase (iNOS), leading to the increment of nitric oxide (NO) synthesis, which is a potent vasodilator. The up‐regulation of adhesion molecules in endothelial cells by cytokines induces the binding of platelets, neutrophils, monocytes and macrophages, which leads to endothelial cell injury. These effector cells release mediators such as free radicals, proteases, oxidants, leukotrienes and prostaglandins. The overt production of nitric oxide and superoxide anions plays an important role in the pathogenesis of septic shock. Nitric oxide reacts with superoxide anions to generate peroxynitrite, leading to severe cytotoxicity 8, 9.

Accumulated evidence indicates that a gender difference exists in the clinical outcome of various inflammatory disorders, such as sepsis 10. It has been suggested that the vasoprotective and anti‐inflammatory effects of oestrogen (17β‐oestradiol; E_2_) are associated with these protective effects 11. Oestrogen replacement provides protection against *V. vulnificus* LPS‐induced endotoxic shock demonstrated by decreased mortality in both gonadectomized male and female rats 12. When subjected to sepsis caused by caecal ligation and puncture 24 hrs after trauma‐haemorrhage, Ovx mice had a significantly higher mortality than pro‐oestrous mice 13. Ovariectomy leads to oestrogen deficiency and exacerbates mortality caused by sepsis. In addition, acute administration of E_2_ can reduce oxidative injury of the liver and ileum during sepsis in male rats 14. By contrast, administrating high levels of exogenous oestrogen augments cell surface toll‐like receptor 4 expression on murine macrophages and exacerbates endotoxic shock susceptibility 15. Thus, giving an appropriate replacement of oestrogen is important in preventing tissue damage by overt immune responses and to provide protection against infection.

E_2_ has been reported to affect various cellular events in reproductive organs by regulation of autophagy 16. Autophagy is an essential biochemical process in which cytoplasmic target material is transported to lysosomes for degradation. It is necessary for the basic maintenance of cellular homoeostasis and has been suggested to confer a protective effect in sepsis 17. Autophagy has been shown to protect against multiple organ dysfunctions caused by sepsis, including hepatic 18, 19, renal 20, 21 and lung 22, 23 injuries, as well as myocardial dysfunction 24. It is worth noting that in mice, the antioxidant enzyme HO‐1‐mediated autophagy protects against hepatocyte cell death and hepatic injury from infection/sepsis 18. Our previous report revealed that up‐regulation of HSP 70 is associated with activation of autophagy, leading to protection against heat stress 25. Therefore, in this study, we evaluated the preventive effect of long‐term E_2_ replacement on endotoxaemia‐induced septic shock and MODS, such as hepatic and renal dysfunction, rhabdomyolysis and coagulopathy in anaesthetized Ovx rats and further investigated whether activation of autophagy, as well as induction of HO‐1 and HSP70 contribute to the beneficial effects of oestrogen.

## Materials and methods

### Animal preparation

Female Sprague Dawley rats were obtained from the BioLASCO Taiwan Co., Ltd. The animals handling was in accordance with the *Guide for the Care and Use of Laboratory Animals* published by the US National Institute of Health (NIH Publication No. 85‐23, revised 1996). All animals were housed at an ambient temperature of 22 ± 1°C and a humidity of 55 ± 5%. This study was approved by the Institutional Animal Care and Use Committee of National Defense Medical Center, Taiwan (Permit Number: IACUC‐13‐015). Rats were anaesthetized with sodium pentobarbital (50 mg/kg, i.p.) and underwent bilateral ovariectomy at 8 weeks of age to produce an oestrogen‐deficient condition. The ovariectomy and sham‐operated surgery were performed as described previously 26.

### Experimental groups

One week after ovariectomy, the rats were divided into four groups: (*i*) *sham* + *saline group*: rats had undergone sham operations. Nine weeks later, rats were given normal saline, no LPS was given to induce sepsis (*n *=* *10); (*ii*) *sham* + *LPS group*: rats had undergone sham operations. Nine weeks later, rats were given LPS to induce sepsis (*n *=* *24); (*iii*) *Ovx* + *LPS group*: rats were Ovx bilaterally. Nine weeks later, rats were given LPS to induce sepsis (*n *=* *28); (*iv*) *Ovx* + *E*_*2*_ + *LPS group*: Ovx rats were injected with E_2_ (50 μg/kg, s.c., three times a week; Sigma Chemical Co., St. Louis, MO, USA) for 8 weeks beginning 1 week after ovariectomy (*n *=* *28). The ovariectomy time period and dose of E_2_ were based on the findings of previous studies 26, 27.

### LPS‐induced experimental sepsis

After 8‐weeks treatment, rats were re‐anaesthetized with intraperitoneal pentobarbital sodium (30 mg/kg) and urethane (0.5 g/kg). E_2_ was not administered on the day of the LPS experiment. The left carotid artery and vein were cannulated to monitor the changes of blood pressure and administration of LPS or normal saline, respectively. Severe experimental sepsis was induced by sustained infusion of 30 mg/kg LPS (*Escherichia coli*, serotype 0127: B8; Sigma‐Aldrich, St. Louis, MO, USA) diluted in 9 ml normal saline for a maximum of 4 hrs 28. The haemodynamic changes (blood pressure, heart rate, left ventricular pressure) were monitored in real‐time throughout the experimental period. When mean arterial blood pressure was below 40 mmHg, rats were killed by intravenous injection of pentobarbital (30 mg/kg). The survival rate in each group was evaluated. Plasma levels of glutamic–pyruvic transaminase [GPT] (hepatic function index), creatinine [CRE] (renal function index), creatine phosphokinase [CPK] and myoglobin (rhabdomyolysis indicators) were observed at 0, 4 and 6 hrs after LPS initiation. All these biochemical variables were determined using a Fuji DRI‐CHEM 3030 analyzer (Fuji Photo Film, Tokyo, Japan). Six hours after saline or LPS initiation, animals were killed under deep anaesthesia, which was induced by additional pentobarbital (15 mg/kg, i.v.). Hearts and other organs were collected immediately for further *ex vivo* studies.

### Coagulation and fibrinolysis function parameters and cytokine measurements

To observe the coagulation changes of LPS‐induced DIC in rats, platelet counts and prothrombin time were determined 29. Blood (0.5 ml) was withdrawn from the cervical artery into plastic syringes at 0, 4 and 6 hrs. The procedure was performed as described previously 30. Plasminogen activator inhibitor‐1 (PAI‐1) was determined as a marker of fibrinolysis 29. Plasma PAI‐1 levels were determined by a commercial ELISA kit (IMUCLONE rat PAI‐1, American Diagnostica Inc., Stamford, CT, USA). In addition, plasma levels of TNF‐α and IL‐6 collected at 2, and 4 hrs after LPS initiation were determined using ELISA kits for rat TNF‐α and IL‐6 (R&D System Inc., Minneapolis, MN, USA).

### Measurements of cardiac contractile function

*In vivo* left ventricular function, including left ventricular developed pressure (LVDP) and ±dP/dt, was evaluated to determine changes in cardiac function during endotoxaemia. A catheter containing a fiber optic miniature pressure sensor (OPP‐M25, OpSens, Quebec, Canada) was inserted *via* the right carotid artery into the left ventricle to determine the real‐time left ventricular pressure and the derivatives ±dP/dt during the experimental period. A Powerlab data acquisition system (ADInstruments, Colorado Springs, CO, USA) was used to acquire and analyse measurements. LVDP was shown at 4 hrs after LPS initiation and presented as a percentage of the baseline.

### Superoxide anions determination

Skeletal muscle samples were taken from the left gastrocnemius muscle 6 hrs after LPS initiation to determine superoxide anion levels. Superoxide anion production is measured by lucigenin‐derived chemiluminescence. The method used was as described previously 31. Briefly, skeletal muscle samples (150–200 mg) were placed in 37°C Krebs‐HEPES buffer and allowed to equilibrate for 10 min. Scintillation plates containing Krebs‐HEPES buffer were placed into a microplate luminometer (Hidex, Microplate Luminometer, Turku, Finland). After recording background counts, a skeletal muscle sample was added to each well and incubated with 50 μl lucigenin (125 μM) for 1 min. Counts were then recorded for each well, and the respective background was subtracted. All samples were dried in a drying cabinet for 2 weeks for expressing results on a milligram skeletal muscle dry weight basis. These results were expressed as counts per second (cps) per milligram dry weight of tissues.

### Determination of glutathione levels in cardiomyocytes

The levels of reduced form (GSH) and oxidized form (GSSG) of glutathione in the myocardium were measured 6 hrs after LPS initiation to reflect the *in vivo* oxidative stress 31, using a GSH detection kit (Enzo Life Sciences, Farmingdale, NY, USA) according to the manufacturer's instructions.

### Western blot analysis

The left ventricular myocardium was isolated 6 hrs after LPS initiation to detect inflammation‐related proteins iNOS and NF‐κB p65, autophagy marker proteins Atg12 32 and light chain 3B (LC3B) 18, HO‐1 and HSP70 expression by Western blotting, which was performed as described previously 31. The primary antibodies used in this experiment were mouse monoclonal anti‐iNOS (1:2000; BD Transduction Laboratories, Lexington, KY, USA), mouse monoclonal anti‐HO‐1 (1:2000; Santa Cruz Biotechnology, Santa Cruz, CA, USA), mouse monoclonal anti‐HSP70 (1:3000; Assay Designs, Farmingdale, NY, USA), rabbit polyclonal NF‐κB p65 antibody (1:2000; Millipore, Bedford, MA, USA), rabbit polyclonal Atg12 (1:2000; Cell Signaling, Danvers, MA, USA) and rabbit polyclonal LC3B (1:2000; Cell Signaling). The ratios of detected proteins to α‐actin and LC3B‐II to LC3B‐I were calculated to standardize densitometry measurements between individual samples.

### Immunohistochemistry for detection of myoglobin deposition in renal tubule cells

The kidneys were fixed in phosphate‐buffered formalin, embedded in paraffin and sectioned at 6 μm. Immunostaining was performed with an antibody against myoglobin (1:400, Dako, Carpinteria, CA, USA) and then with biotinylated secondary antibody (Dako) and avidin‐biotin‐peroxidase complex (Dako). The slides were counterstained lightly with haematoxylin. The immunoreactivity was screened semi‐quantitatively by randomly observing the number of myoglobin contained tubules in 15 high power fields of a whole section and estimating the proportion of myoglobin contained tubules of total renal tubules in each field.

### Statistical analysis

The data are expressed as group means ± S.E.M. Statistical evaluation was performed with one‐factor analysis of variance (anova) followed by the Newman–Keuls post hoc comparison test. Chi‐square test followed by Fisher's exact test was used for comparison of the survival rate among groups of rats. Statistical evaluation of MAP was performed with two‐way anona followed by the LSD post hoc comparison test. A *P* value of < 0.05 was deemed statistically significant.

## Results

### Plasma levels of E_2_

Plasma E_2_ levels were determined at the end of the experimental endotoxaemia (9 weeks after ovariectomy). The plasma E_2_ concentration was 70.9 ± 7.1 and 67.82 ± 3.87 pg/ml in rats of sham + saline and sham + LPS groups, respectively. The plasma E_2_ concentration of the Ovx + LPS group was 34.08 ± 5.1 pg/ml, which is significantly lower than that of the sham + saline group (*P *<* *0.05). After long‐term treatment of E_2_ (50 μg/kg, s.c., 3 times/week), the plasma level of E_2_ was 64.86 ± 4.80 pg/ml in the Ovx + E_2_ + LPS group, which is significantly higher than that of the Ovx + LPS group (*P *<* *0.05), but not significantly different from that of the sham + saline group (*P *>* *0.05).

### Survival rate

All rats in the sham + saline group survived until the end of the experiment. The survival rate was reduced to 66.7% in the sham + LPS group, which is almost significantly lower than that of the sham + saline group (*P *=* *0.07). Administering LPS to Ovx rats resulted in endotoxaemia high mortality. The survival rate in the Ovx + LPS group was 32.1%, which is significantly lower than the sham + saline and sham + LPS groups (*P *<* *0.05). Long‐term replacement with E_2_ increased the survival rate of Ovx rats in endotoxaemia (Ovx + E_2_ + LPS: 60.7%), which did not show a statistical difference when compared with the Ovx + LPS group and was not significantly different from the sham + LPS group (*P *>* *0.05) (Table 1).

**Table 1:** Effects of oestrogen on the survival rate of Ovx rats with endotoxaemia

Groups	Survival rate % (Survivors/Total rats)
Sham + saline	100.0
	(10/10)
Sham + LPS	66.7
	(16/24)
Ovx + LPS	32.1* ^,^ #
	(9/28)
Ovx + E_2_ + LPS	60.7*
	(17/28)

Female rats were sham‐operated or ovariectomized (Ovx) for 9 weeks, followed by LPS administration (30 mg/kg/4 hrs, i.v.) to induce endotoxaemia. In the Ovx + E_2_ + LPS group, Ovx rats were treated with 17β‐Oestradiol (E_2_; 50 μg/kg) subcutaneously three times per week for 8 weeks. The survival rate was calculated 6 hrs after initiation of LPS administration.

*P <* *0.05 *versus* Sham + saline; ^#^ *P *<* *0.05 *versus* Sham + LPS.

### Effects of E_2_ on haemodynamic changes in Ovx rats with endotoxaemia

**Figure 1:**
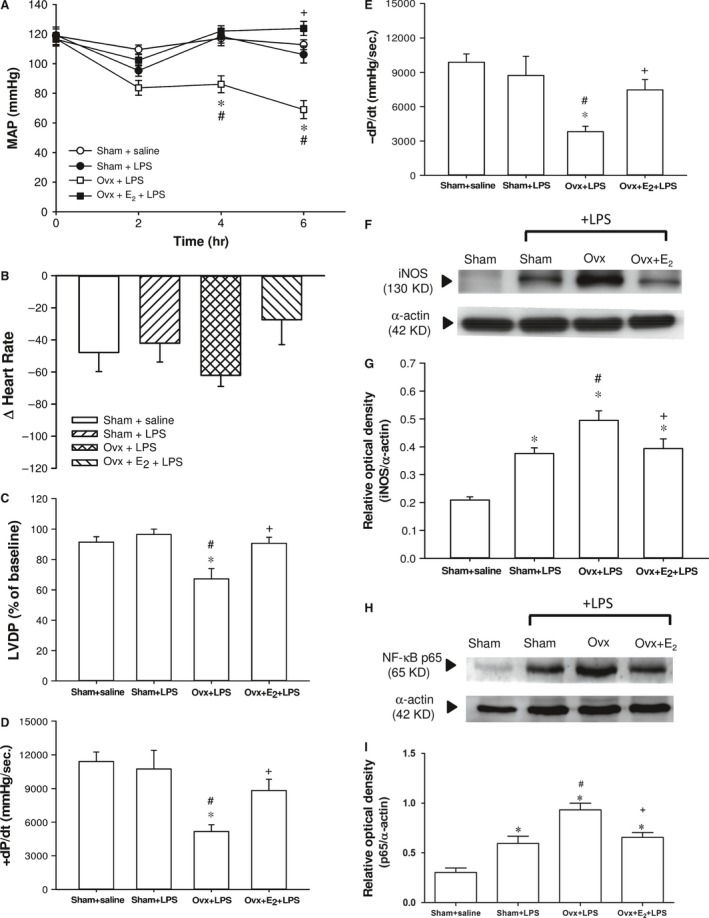
The effect of long‐term treatment with 17β‐oestradiol (E_2_) on haemodynamic changes and cardiac function in ovariectomized (Ovx) rats with endotoxaemia induced by lipopolysaccharide (LPS) infusion (30 mg/kg, i.v.) for 4 hrs. Ovx rats were treated with E_2_ (50 μg/kg) subcutaneously three times per week for 8 weeks, followed by LPS infusion. **A**: mean arterial blood pressure (MAP), **B**: changes in heart rate from 0 to 6 hrs after LPS initiation, **C**: left ventricular developed pressure (LVDP), **D** & **E**: +dP/dt and −dP/dt of LVDP, **F**: representative Western blot of iNOS in left ventricle; **G**: relative optical density (iNOS/α‐actin); **H**: representative Western blot of NF‐κB p65 in left ventricle; **I**: relative optical density (p65/α‐actin); Values are expressed as mean ± S.E.M.; *P <* *0.05 *versus* Sham + saline; ^**#**^ *P *<* *0.05 *versus* Sham + LPS; ^**+**^ *P *<* *0.05 *versus* Ovx + LPS; n = 9–17.

The baseline values for mean arterial blood pressure (MAP) and heart rate did not show significant differences among groups. The MAP of sham + saline group did not significantly change during the experimental period. MAP dropped gradually when LPS infusion was initiated. Surprisingly, after a 4‐hr LPS infusion, the MAP of the sham + LPS group did not significantly reduce when compared with the sham + saline group, and this lasted for 6 hrs. The MAP of Ovx rats gradually dropped and were significantly lower than that of sham + LPS group 4–6 hrs after LPS initiation (*P *<* *0.05). Long‐term E_2_ replacement in Ovx rats prevented the hypotension caused by LPS when compared with the Ovx + LPS group (*P *<* *0.05) (Fig. 1A). The changes of heart rate were not significantly different among groups throughout the experiment (Fig. 1B). The LVDP (Fig. 1C) and average ±dP/dt (Fig. 1D and E) were determined to evaluate cardiac contractile function. In the sham + LPS group, the LVDP and ±dP/dt remained unchanged by a 4‐hrs infusion of LPS, whereas they were significantly reduced in the Ovx + LPS rats (*P *<* *0.05). Long‐term replacement of E_2_ in Ovx rats improved these parameters during endotoxaemia when compared with the rats of the Ovx + LPS group (*P *<* *0.05).

### ***Effects of E***_***2***_ ***on cardiac inflammation‐related proteins iNOS and NF‐***κ***B p65 expression in Ovx rats with endotoxaemia***

The LPS challenge caused increased levels of iNOS and NF‐κB p65 in left ventricles at 6 hrs. The levels of cardiac iNOS and p65 protein expression of the three LPS‐challenged groups were significantly higher than that of the sham + saline group (Fig. 1F, G, H & I). The elevation of cardiac iNOS and p65 in the Ovx + LPS group was significantly higher than in the sham + LPS group (*P *<* *0.05). The long‐term replacement of E_2_ in Ovx rats prevented the rise in cardiac iNOS and p65 protein expression by LPS, with levels similar to those of the sham + LPS group.

### Effects of E_2_ on cardiac autophagy marker proteins Atg12 and LC3B expression in Ovx rats with endotoxaemia

**Figure 2:**
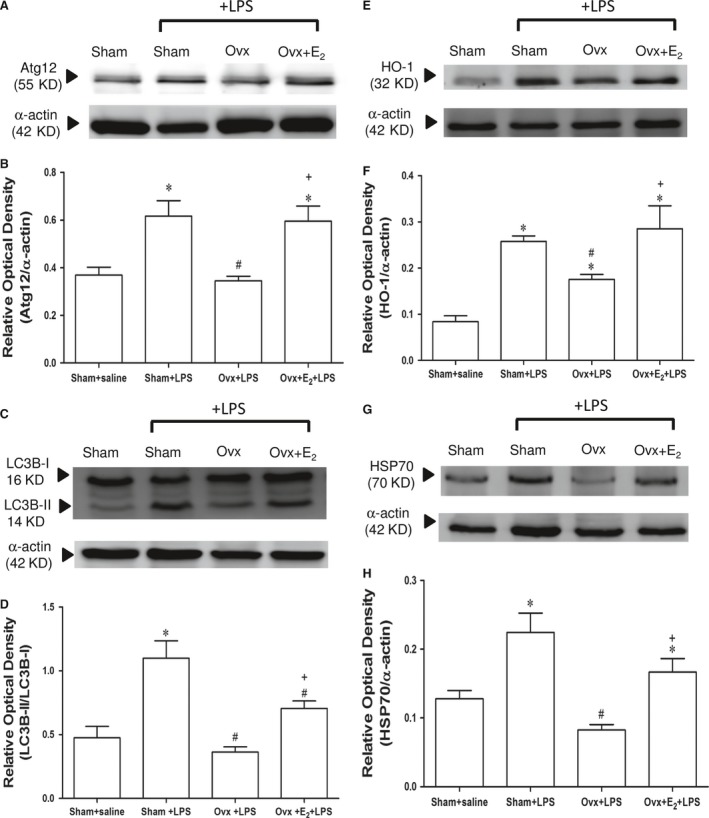
The effect of long‐term treatment with 17β‐oestradiol (E_2_) on cardiac autophagy marker proteins, HO‐1 and HSP70 levels in ovariectomized (Ovx) rats with endotoxaemia induced by lipopolysaccharide (LPS) infusion (30 mg/kg, i.v.) for 4 hrs. Ovx rats were treated with E_2_ (50 μg/kg) subcutaneously three times per week for 8 weeks, followed by LPS infusion. **A**: representative Western blot of Atg12 in left ventricle; **B**: relative optical density (Atg12/α‐actin); **C**: representative Western blot of LC3B in the left ventricle; **D**: relative optical density (LC3B‐II/LC3B‐I); **E**: representative Western blot of HO‐1 in the left ventricle; **F**: relative optical density (HO‐1/α‐actin); **G**: representative Western blot of HSP70 in the left ventricle; **H**: relative optical density (HSP70/α‐actin);values are expressed as mean ± S.E.M.; *P <* *0.05 *versus* Sham + saline; ^**#**^ *P *<* *0.05 *versus* Sham + LPS; ^**+**^ *P *<* *0.05 *versus* Ovx + LPS; n = 8.

Six hours after LPS initiation, the levels of Atg12, LC3B‐I and LC3B‐II increased significantly in the sham + LPS and Ovx + E_2_ + LPS groups compared with the Ovx + LPS group (*P *<* *0.05). The ratios of LC3B‐II levels to LC3B‐I levels in the sham + LPS and Ovx + E_2_ + LPS groups were also higher than the Ovx + LPS group (Fig. 2A, B, C & D) (*P *<* *0.05). Long‐term replacement of E_2_ in Ovx rats raised autophagy‐related proteins in LPS‐stimulated cardiac tissues.

### Effects of E_2_ on cardiac HO‐1 and HSP70 protein expression of Ovx rats with endotoxaemia

LPS challenge significantly stimulated cardiac HO‐1 induction when compared with sham + saline group. The HO‐1 level in the Ovx + LPS group was significantly lower than in the sham + LPS and Ovx + E_2_ + LPS groups (Fig. 2E and F) (*P *<* *0.05). Also, LPS stimulated the elevation of HSP70 protein in the sham + LPS and Ovx + E_2_ + LPS groups at 6 hrs, which was not found in the Ovx + LPS group (Fig. 2G and H).

### Effects of E_2_ on LPS‐induced organ dysfunction in Ovx rats with endotoxaemia

**Figure 3:**
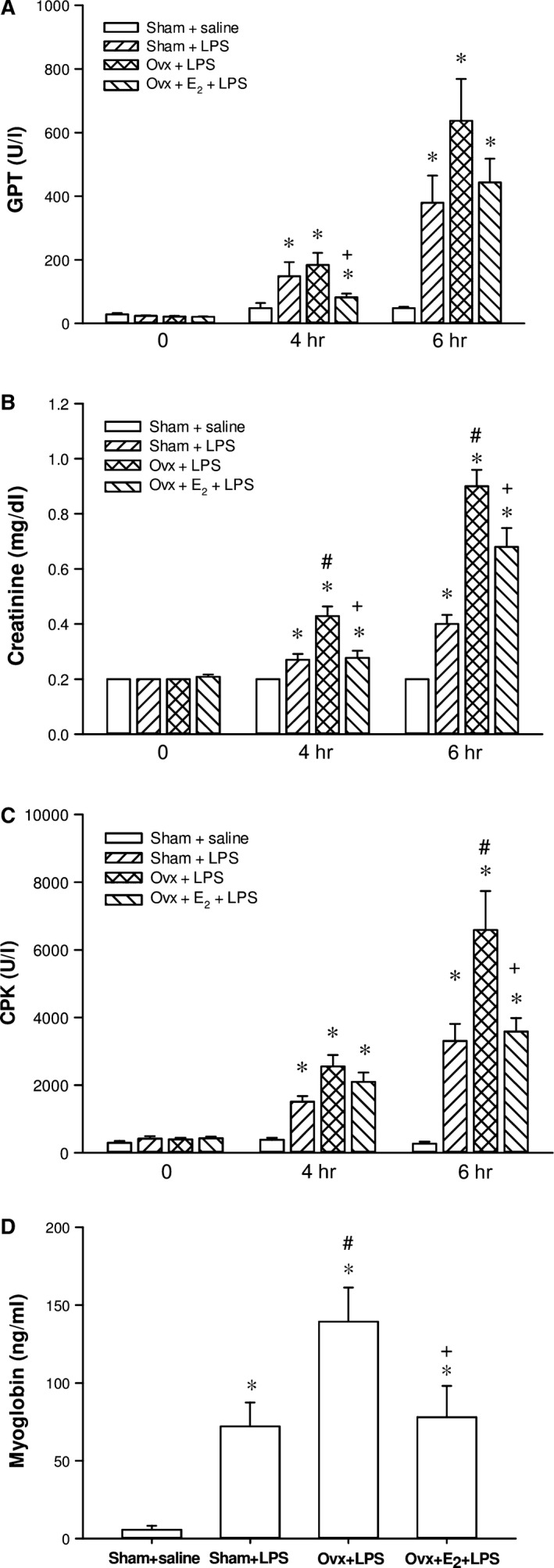
The effect of long‐term treatment with 17β‐oestradiol (E_2_) on hepatic and renal dysfunction, and rhabdomyolysis in ovariectomized (Ovx) rats with endotoxaemia induced by lipopolysaccharide (LPS) infusion (30 mg/kg, i.v.) for 4 hrs. Ovx rats were treated with E_2_ (50 μg/kg) subcutaneously 3 times per week for 8 weeks, followed by LPS infusion. **A**: plasma level of glutamic–pyruvic transaminase (GPT), **B**: creatinine, **C**: creatine phosphokinase (CPK), **D**: myoglobin; Values are expressed as mean ± S.E.M.; *P <* *0.05 *versus* Sham + saline; ^**#**^ *P *<* *0.05 *versus* Sham + LPS; ^**+**^ *P *<* *0.05 *versus* Ovx + LPS; n = 9–17.

After initiation of the LPS infusion, plasma levels of GPT, CRE, CPK and myoglobin increased progressively in the three LPS‐challenged groups, all were significantly higher than in the sham + saline group at 4 and/or 6 hrs. Rats with an oestrogen deficiency induced by the ovariectomy (Ovx + LPS group) showed significantly higher plasma levels of CRE, CPK and myoglobin than those of the sham + LPS group at 6 hrs (*P *<* *0.05), and this was prevented by the long‐term replacement of E_2_ (Ovx + E_2_ + LPS group) (*P *<* *0.05). E_2_ also prevented the elevation of GPT levels at 4 hrs when compared with the Ovx + LPS group (*P *<* *0.05) (Fig. 3A, B, C & D).

### Effects of E_2_ on glutathione levels in myocardium and superoxide anion production in skeletal muscles of Ovx rats with endotoxaemia

**Figure 4:**
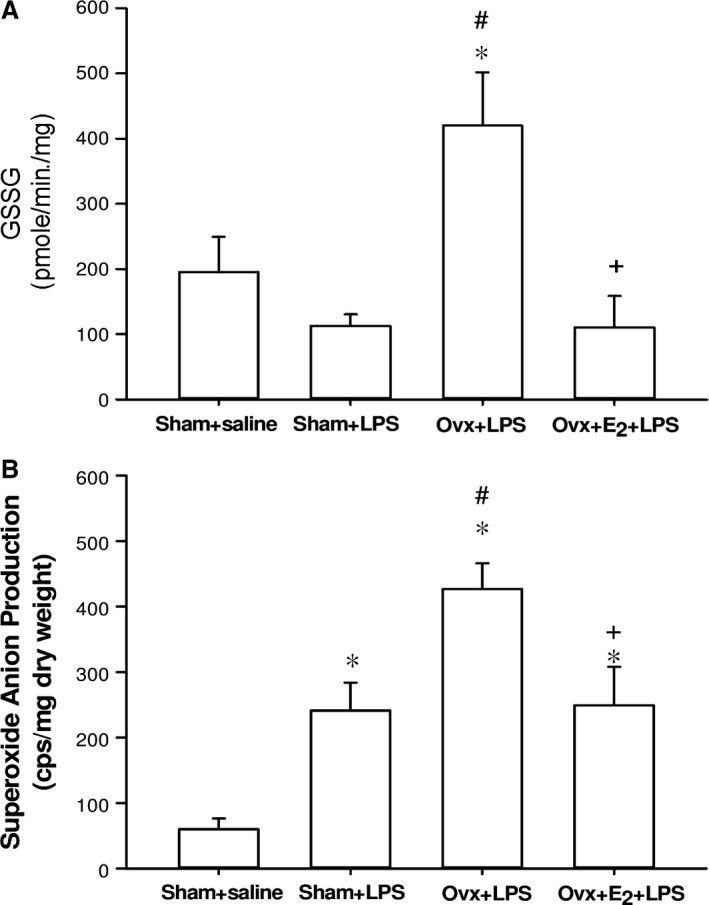
The effect of long‐term treatment with 17β‐oestradiol (E_2_) on oxidized glutathione (GSSG) in the heart (**A**) and superoxide anion production of skeletal muscle (**B**) in ovariectomized (Ovx) rats with endotoxaemia induced by lipopolysaccharide (LPS) infusion (30 mg/kg, i.v.) for 4 hrs. Values are expressed as mean ± S.E.M.; *P <* *0.05 *versus* Sham + saline; ^**#**^ *P *<* *0.05 *versus* Sham + LPS; ^**+**^ *P *<* *0.05 *versus* Ovx + LPS; n = 6–9.

The GSSG (oxidized glutathione) levels in the hearts of the Ovx + LPS group were significantly higher than those of the sham + LPS group. Long‐term of E_2_ in Ovx rats markedly reduced GSSG levels at 6 hrs after LPS initiation when compared with the Ovx + LPS group (*P *<* *0.05) (Fig. 4A). However, there was no significant difference in reduced form of glutathione (GSH) among groups (data not shown).

The levels of superoxide anion production in gastrocnemius muscle were significantly elevated 6 hrs after LPS initiation in the three LPS‐challenged groups when compared with the sham + saline group. The increase in superoxide anion production in the Ovx + LPS group was significantly higher than that of the sham + LPS group, whereas it was prevented by the long‐term replacement of E_2_ (Fig. 4B). The level of superoxide anion production in the Ovx + E_2_ + LPS group was not significantly different from that of the sham + LPS group (*P *>* *0.05).

### Effects of E_2_ on myoglobin deposition in renal tubules in Ovx rats with endotoxaemia

**Figure 5:**
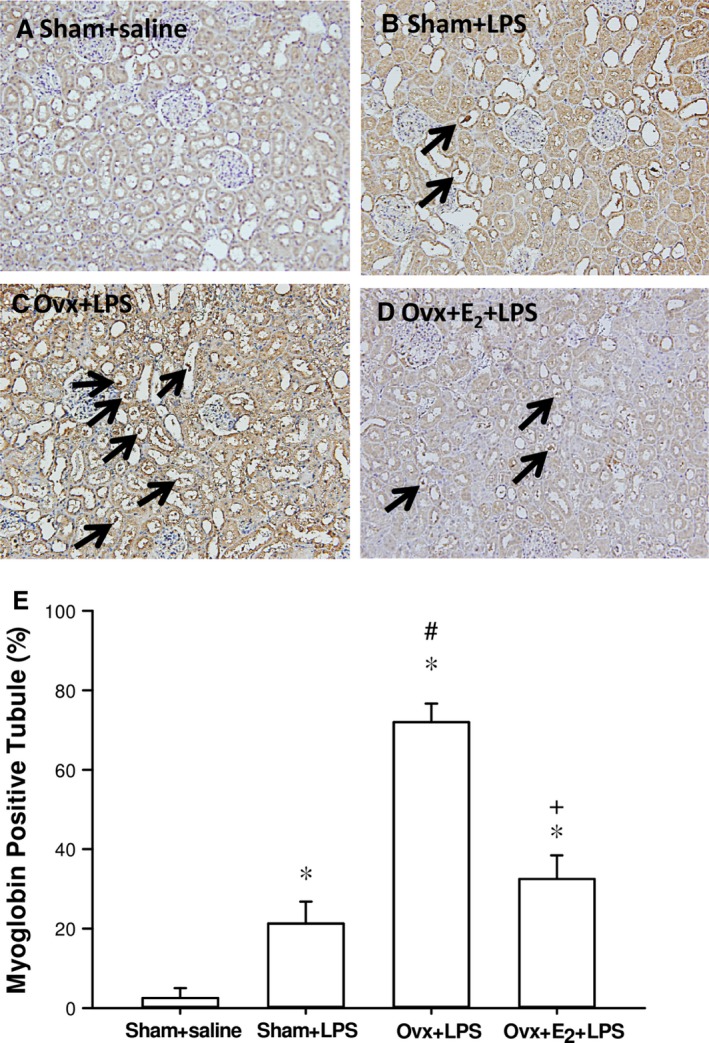
Effects of long‐term treatment with 17β‐oestradiol (E_2_) on immunohistochemical staining of myoglobin in kidneys 6 hrs after lipopolysaccharide (LPS) initiation in ovariectomized (Ovx) rats. Histologic sections from the Sham + saline group (**A**), Sham + LPS group (**B**), Ovx + LPS group (**C**), Ovx + E_2_ + LPS group (**D**), stained with myoglobin antibody (magnification, ×200). The arrows show the development of myoglobin casts. (**E)** Averaged per cent of myoglobin‐positive renal tubules. Data expressed as mean ± SEM; n = 3–5; *P <* *0.05 *versus* Sham + saline; ^#^ *P *<* *0.05 *versus* Sham + LPS; ^+^ *P *<* *0.05 *versus* Ovx + LPS; n = 5.

The sham + saline group showed negative myoglobin immunostaining in the renal tubules of all rats. In the sham + LPS group, LPS caused a significant increase in myoglobin deposition in the renal tubules compared with the sham + saline group. The increase in myoglobin‐containing renal tubules in the Ovx + LPS group was significantly higher than that of the sham + LPS group (*P < 0.05*). Long‐term treatment with E_2_ significantly reduced myoglobin‐containing renal tubules in Ovx rats with endotoxaemia (Fig. 5).

### Effects of E_2_ on the coagulation function in Ovx rats with endotoxaemia

**Figure 6:**
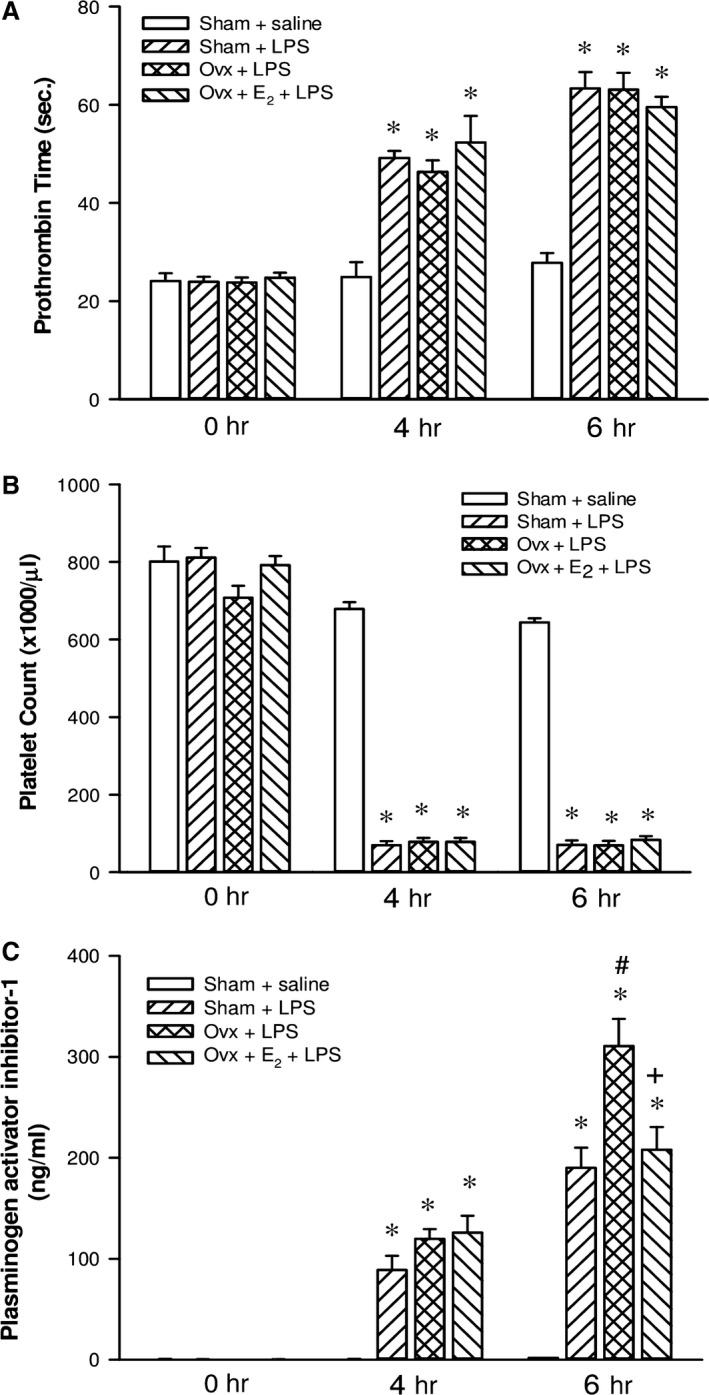
The effect of long‐term treatment with 17β‐oestradiol (E_2_) on coagulopathy in ovariectomized (Ovx) rats with endotoxaemia induced by lipopolysaccharide (LPS) infusion (30 mg/kg, i.v.) for 4 hrs. Ovx rats were treated with E_2_ (50 μg/kg) subcutaneously three times per week for 8 weeks followed by LPS infusion. **A**: prothrombin time; **B**: platelet count; **C**: plasma levels of plasminogen activator inhibitor‐1. Values are expressed as mean ± S.E.M.; *P <* *0.05 *versus* Sham + saline; ^**#**^ *P *<* *0.05 *versus* Sham + LPS; ^**+**^ *P *<* *0.05 *versus* Ovx + LPS; n = 9–17.

As shown in Fig. 6A and B, there was no significant difference in baseline prothrombin time and platelet count among all groups. The prothrombin time value and platelet count of the sham + saline group did not show significant changes during the experimental period. After a 4‐hr LPS infusion, prothrombin time was markedly prolonged, accompanied with an evident decrease in the platelet count in the three LPS‐challenged groups, which did not improve by the long‐term replacement of E_2_. Furthermore, after LPS administration, PAI‐1 concentration was noted to increase with time. Plasma levels of PAI‐1 were dramatically elevated in the three LPS‐treated groups at 4 and 6 hrs (Fig. 6C). A significantly higher level was shown in the Ovx + LPS group than in the sham + LPS group at 6 hrs (*P *<* *0.05). The long‐term replacement of E_2_ in Ovx rats significantly reduced the plasma level of PAI‐1, when compared with that of the Ovx + LPS group (*P *<* *0.05).

### Effects of E_2_ on proinflammatory cytokines in Ovx rats with endotoxaemia

**Figure 7:**
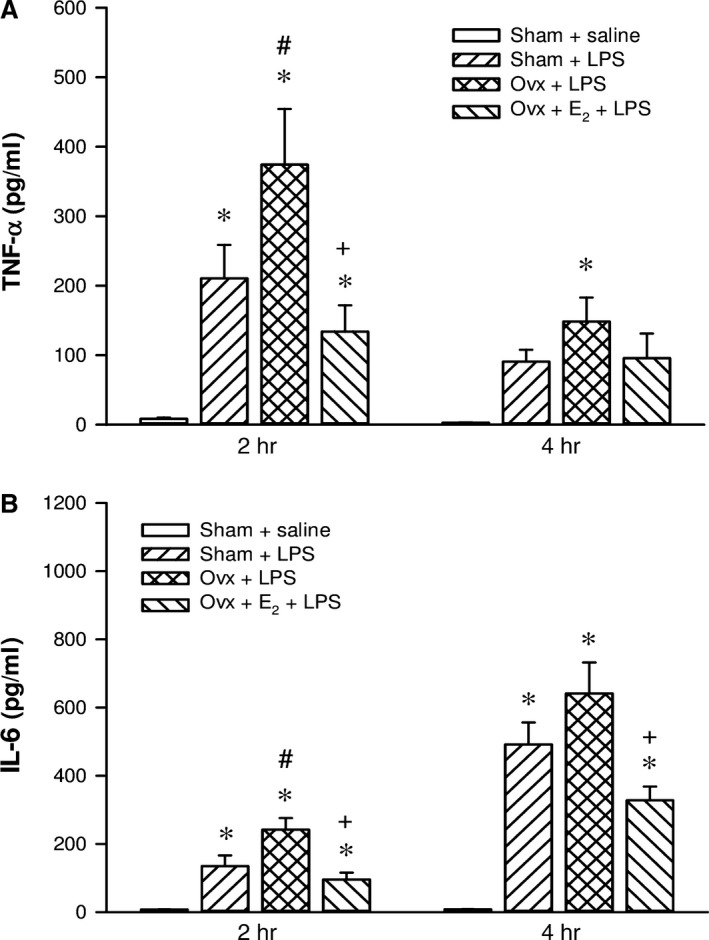
The effect of long‐term treatment with 17β‐oestradiol (E_2_) on plasma levels of TNF‐α (**A**) and IL‐6 (**B**) in rats with endotoxaemia induced by lipopolysaccharide (LPS) infusion (30 mg/kg, i.v.) for 4 hrs. Ovx rats were treated with E_2_ (50 μg/kg) subcutaneously three times per week for 8 weeks, followed by LPS infusion. Values are expressed as mean ± S.E.M.; *P <* *0.05 *versus* Sham + saline; ^**#**^ *P *<* *0.05 *versus* Sham + LPS; ^**+**^ *P *<* *0.05 *versus* Ovx + LPS; n = 9–17.

After initiation of the LPS infusion, plasma levels of TNF‐α and IL‐6 markedly increased in the three LPS‐challenged groups, all of them were significantly higher than that of the sham + saline group (*P *<* *0.05). Two hours after LPS initiation, concentrations of both cytokines in the Ovx + LPS group were significantly higher than those of the sham + LPS group (*P *<* *0.05). Long‐term replacement of E_2_ in Ovx rats prevented the rise in TNF‐α and IL‐6 concentrations at 2 hrs, when compared with the Ovx + LPS group (*P *<* *0.05). The inhibitory effect of exogenous E_2_ on IL‐6 release lasted up to 4 hrs after LPS initiation (Fig. 7).

## Discussion

In the present study, we demonstrate that endogenous oestrogens and the long‐term replacement of E_2_ play an important role in preventing septic shock, and ameliorating multiple organ dysfunction syndrome in endotoxaemia, including hepatic, renal, and fibrinolysis dysfunction, and skeletal damage. Oestrogen deficiency, induced by ovariectomy, increased the severity caused by endotoxaemia. Interestingly, sham‐operated rats, with a normal physiological concentration of endogenous oestrogens, presented a resistance to damage caused by endotoxaemia on the cardiovascular system, demonstrated by maintenance of blood pressure and cardiac contractile function, which was not observed in endotoxaemic male rats 30. The antioxidant and anti‐inflammatory effects of oestrogen are involved in these protective effects. Activation of autophagy and induction of HO‐1 and HSP70 may be the cellular mechanisms of action that contribute to the protective effects of oestrogen.

Oestrogen is a potent steroid hormone present in high levels in females from adolescence to menopause and in low levels in men. Women have a better outcome during bacterial infections and have a lower risk of developing sepsis compared with men 33. Thus, sex hormones, especially oestrogens have been suggested to play an important role in the beneficial effect. It has been shown that oestrogen has anti‐inflammatory effects on microglia and macrophages activation by LPS 34, 35, 36, 37. On the contrary, oestrogen has been reported to enhance proinflammatory responses of macrophages to TLR4 activation by LPS 38, 39 and is required for a proper immune response to bacterial and viral pathogens in the female brain 40. Rettew *et al*. also found that oestrogens possess immune‐enhancing effects *via* an increase in cell surface TLR4 expression on macrophages. High levels of exogenous oestrogen resulted in a marked increase in LPS‐associated morbidity 15. These reports indicate that oestrogen possesses an immune‐modulating capacity. In *in vivo* circumstances, oestrogen can promote the activation of immune cells to prevent pathogen infection. Whereas, after a long period of oestrogen insufficiency, oxidative stress significantly increased 41, which may trigger more inflammatory responses when challenged by LPS, leading to a higher concentration of cytokine release. In the present study, ovariectomy has been conducted to induce a significant reduction in oestrogen level. Over a long period (9 weeks), inflammatory responses (cytokine release) and organ dysfunction were enhanced, accompanied with a significantly lower survival rate after LPS challenge. Replacement with E_2_, the major oestrogen produced by the ovaries, almost attenuated the severity of organ dysfunction amplified by ovariectomy. This evidence indicates that endogenous oestrogen, mainly E_2_, indeed contributes to a beneficial outcome during endotoxaemia. The anti‐inflammatory and antioxidant effects offered by oestrogen are associated with the *in vivo* protection. Furthermore, based on the immune‐enhancing effect of E_2_, we suggest that under endotoxaemia or sepsis, acute administration of exogenous oestrogen is not suitable for females with physiologically endogenous E_2_. This may result in overstimulation of inflammatory responses, leading to increases in the severity of organ dysfunction and septic shock mortality.

The preventive effect of oestrogen on endotoxaemia‐induced circulatory failure is extremely pronounced when compared with oestrogen‐insufficient Ovx rats. In our previous study, male rats given LPS 30 mg/kg infusion for 4 hrs can cause marked hypotension (septic shock) at 6 hrs 30. By contrast, the blood pressure of female rats (Sham + LPS & Ovx + E_2_ + LPS groups) was not significantly changed by LPS in this study, indicating that oestrogen produced excellent cardio‐ and vasoprotective effects during sepsis. It is well known that oestrogen can provide a vasoprotective effect *via* modulating injury‐induced inflammation, and oxidative stress in arteries and isolated vascular smooth muscle cells. Oestrogen, *via* oestrogen receptor activation, can inhibit the expression of the proinflammatory mediator TNF‐α and inhibit NF‐κB signalling by a variety of mechanisms to provide vascular protection 42. Furthermore, we also found oestrogen prevented endotoxaemia‐induced expression of iNOS in the myocardium, which can produce high levels of nitric oxide. It is responsible for direct effects on vascular tone, depression of mitochondrial respiration and further release of proinflammatory cytokines, leading to myocardial depression 43. Nitric oxide reacts with superoxide anions to generate a cytotoxic product, peroxynitrite 9, also contributing to organ dysfunction. Therefore, in this study, the cardiovascular protection of oestrogen during sepsis can be mediated *via* the inhibitory effects on cellular NF‐κB signalling activation, leading to TNF‐α and IL‐6 release and cardiac iNOS induction.

Autophagy has been shown to protect against multiple organ dysfunction caused by sepsis 17. In this study, LPS‐activated autophagy was augmented in sham‐operated rats (sham + LPS group) and Ovx rats with long‐term E_2_ replacement (Ovx + E_2_ + LPS group), indicating that E_2_ preserves the autophagic function in acute inflammation caused by endotoxaemia. The protective effect of E_2_ on endotoxaemia may be associated with activation of autophagy. Autophagy is a well‐conserved and catabolic process in cells involving the vesicular sequestration of cytoplasmic proteins, pathogens or organelles by a double‐membrane vesicle, termed an autophagosome. The fusion of the autophagosome with a lysosome forms an autolysosome, in which the captured materials were degraded into their subcomponents, which can then be recycled 44. Upon extracellular or intracellular stress and signals, autophagy may be markedly up‐regulated to function as a self‐destructive signalling pathway that promotes cell survival in an adverse environment. Recently, it has been shown in mice that HO‐1‐mediated autophagy protects against hepatocyte cell death and hepatic injury from infection/sepsis 18. Furthermore, HSP70 has been suggested to activate the autophagic process to protect against heat‐shock stress 25. In this study, we demonstrated that oestrogen can preserve the capacity of cellular HO‐1 and HSP70 production during endotoxaemia (Fig. 2). HO‐1 and HSP70 have been reported to be cytoprotective proteins that ameliorate injuries from endotoxaemia 45. Therefore, the protective effect of E_2_ can be mediated *via* induction of HO‐1 and HSP70, by which E_2_ may further activate autophagic processes to provide a beneficial effect during endotoxaemia. Nevertheless, in a recent study, E_2_ has been reported to protect cardiomyocytes against LPS‐induced injury by inhibiting autophagy 46. The results were obtained from an *in vitro* study (cultured cardiomyocytes), in which E_2_ was administered acutely (30 min. prior to LPS treatment). By contrast, in the present study, E_2_ was treated with Ovx rats for a long period and showed protective effect on endotoxaemia *in vivo*. Thus, activation of autophagy by E_2_ emerged in myocardium from an animal model of sepsis is more clinically relevant.

Oxidative stress has been reported to be elevated in sepsis 47. It initiates inflammatory responses and cell activation, and increases activation of the redox‐sensitive transcription factor NF‐κB in patients with sepsis 48, 49, 50. Antioxidants have been reported to possess beneficial effects in sepsis 51. Oestrogen attenuates oxidative stress by preventing the generation of reactive oxygen species (ROS) and by scavenging ROS in the myocardium and in the vasculature 52. Therefore, the attenuation of oxidative stress by oestrogen demonstrated by reduction of superoxide anion production and suppression of oxidized form of glutathione (GSSG) formation (Fig. 4) may contribute to the prevention of MODS in endotoxaemia.

Sepsis is often associated with DIC 53, 54. Endotoxins, by causing the systemic release of cytokines, may contribute to the development of DIC in patients with infections. Cytokines are mainly produced by activated mononuclear cells and endothelial cells and cause the derangement of the coagulation system in DIC 55. The widespread and ongoing activation of coagulation leads to vascular fibrin deposition. It compromises an adequate blood supply to various organs, which can result in organ failure 56. Because of ongoing activation of the coagulation system, exhaustion of coagulation and platelets may occur. This situation could result in serious bleeding. Thrombosis and bleeding could concurrently occur, resulting in a difficult problem for the clinician. Furthermore, endotoxaemia can result in a rapidly occurring increase in fibrinolytic activity, most probably caused by the release of plasminogen activators from endothelial cells. This profibrinolytic response is almost immediately followed by a suppression of fibrinolytic activity because of a sustained increase in plasma levels of PAI‐1 57, 58. In this study, LPS induced a significant reduction in platelet count and prolongation of prothrombin time. Oestrogen did not prevent the activation of the coagulation system (Fig. 5A and B), whereas the inhibition of fibrinolytic activity was ameliorated *via* reduction in PAI‐1 production (Fig. 5C). The improvement of fibrinolysis function helps to remove formed thrombus and to increase blood perfusion of organs, which may contribute to reducing the severity of MODS. It has been shown that severe organ damage was observed along with the increased plasma concentration of PAI‐1 59. There is a direct relationship between PAI‐1 plasma concentration and the severity of MODS in patients with DIC, which also correlates with a worse prognosis 60, 61. PAI‐1 expression at the protein and mRNA level can be strongly up‐regulated by TNF‐α, IL‐1 or LPS in different cell types 62, 63. Therefore, the inhibitory effect of oestrogen on TNF‐α release participates in suppression of PAI‐1 production in endotoxaemia. However, E_2_ did not show anticoagulant effects under LPS challenge, which may be because hormone therapy has been known to induce changes in haemostasis, and this may increase the risk of venous thromboembolism 64. A significant decrease in inhibitors of coagulation (antithrombin and/or protein C and protein S) and a significant increase in activated protein C resistance or baseline thrombin generation is associated with oral oestrogen use 65. Moreover, in this study, a high dose of LPS was used to induce endotoxaemia, which led to severe coagulopathy even at 2 hrs after LPS initiation. Thus, these may be the reasons why E_2_ did not prevent the overt procoagulation in the study.

Rhabdomyolysis is a common and potentially lethal clinical syndrome that results from acute muscle fibre necrosis with leakage of muscle constituents into the blood. It occurs from inherited diseases, toxins, muscle compression or overexertion or inflammatory processes, among other disorders. Myoglobinuria and deposition of myoglobin in renal tubules are the most significant consequence, leading to acute renal failure in 15–50% of patients with rhabdomyolysis 66. In this study, oestrogen prevented the ovariectomy augmented elevation of plasma CPK, myoglobin and myoglobin accumulation in renal tubules, indicating that oestrogen provides protection against LPS‐induced rhabdomyolysis and may contribute to improvement of renal function.

## Conclusions

Long‐term oestrogen deficiency increases susceptibility to endotoxaemia or infection, leading to enhancement of inflammatory responses and MODS, which can be prevented by endogenous oestrogen or replacement with E_2_. Oestrogen reduces the severity of circulatory failure, renal dysfunction, rhabdomyolysis and fibrinolysis dysfunction in severe sepsis, which are associated with its anti‐inflammatory and antioxidant effects. Activation of autophagy, as well as induction of HO‐1 and HSP70, may be the cellular mechanisms of action that contribute to the protective effects of oestrogen. The beneficial effect of oestrogen on immune modulation has the potential to prevent infection or inflammatory diseases after menopause. Long‐term replacement of oestrogen may increase resistance to bacterial infection and reduce the severity of sepsis in post‐menopausal women.

## Conflict of interest

The authors confirm that there is no conflict of interests.
